# Application of Aptamer–Carbon Surfaces for Electrochemical Label-Free Detection of Vancomycin

**DOI:** 10.3390/bios16070353

**Published:** 2026-06-24

**Authors:** Izabela Zaras, Piotr Pieta, Marta Jarczewska

**Affiliations:** 1 Chair of Medical Biotechnology, Faculty of Chemistry, Warsaw University of Technology, Noakowskiego 3, 00-664 Warsaw, Poland; izabela.zaras.dokt@pw.edu.pl; 2 Institute of Physical Chemistry, Polish Academy of Sciences, Kasprzaka 44/52, 01-224 Warsaw, Poland; ppieta@ichf.edu.pl

**Keywords:** vancomycin, aptamer, carbon-based electrodes, redox indicator

## Abstract

Gold is considered the most widely used surface for the development of aptamer-based layers. However, its high cost, laborious surface-cleaning protocols, and susceptibility of receptor layers to degradation in complex samples, including biological fluids, enforce the search for alternative transducers. One solution is the application of carbon materials, which are inexpensive and allow for the use of a wide potential range when electrochemical measurements are performed. Herein, we present studies on the elaboration of aptamer receptor layers formed on carbon macroelectrodes. To achieve this, a one-step procedure for aptamer molecules containing a pyrene or anthracene group at the 5′ end was used, with immobilization via adsorption facilitated by Π–Π interactions between the anchor group and the carbon surface. It was evidenced that using anthracene-modified aptamer and sodium anthraquinone-2-sulfonic acid (AQMS) redox indicator enabled the detection of a model analyte–vancomycin below the millimolar concentration range. It was also revealed that vancomycin can be successfully detected in serum samples, and the aptasensor exhibits good selectivity towards vancomycin. The latter was observed by comparison of responses in PBS containing solely vancomycin and a solution spiked with vancomycin and a mixture of antibiotics.

## 1. Introduction

Nucleic acids (NA) are one of the most explored biomolecules. Since they are not solely considered the genetic information storage units, their possible application might refer to areas such as therapy and drug delivery systems, as well as diagnostics. The latter scope can be realized by employing NA as receptor elements in biosensors [1]. Particular attention is given to DNA and RNA aptamers, which undergo a conformational switch upon interaction with targets, including metal cations, small organic compounds, proteins, and cells [2,3,4]. To provide efficient binding between the aptamer layer and the target molecule, it is necessary to establish conditions for transducer functionalization that maintain stability while executing an experiment and biosensor storage [5].

Over the years, gold-based substrates became one of the most exploited transducer materials for the development of aptamer-based biosensors. This is justified by the simplicity of Au surface modification, which is currently realized using disulfide/thiol-based aptamers, adenine, and phosphorothioated (PTO) anchors [6,7,8] as well as a cysteamine intermediate layer [9]. It should be noted that the application of gold requires mechanical, chemical, and electrochemical cleaning steps, followed by immediate surface functionalization to prevent the adsorption of impurities from air and solutions. Further minimization of nonspecific adsorption, which is likely to occur, especially when sensors are tested in complex matrices such as blood, can be achieved by immobilizing blocking agents, including alkanethiols or proteins [10,11]. Gold, thanks to its biocompatibility, can be applied in vivo; however, receptor layers formed on such surfaces are prone to degradation as naturally occurring compounds containing sulfur like cysteine might compete for binding to the Au surface [12]. Furthermore, when electrochemical detection methods are combined with sensors, a defined potential window must be used to avoid desorption of the sensing layer. Finally, the high cost of gold might stand against its application for large scale production.

That is the reason for searching for other materials, and one of the options is the utilization of carbon surfaces such as glassy carbon (GC), pyrolytic graphite (edge plane pyrolytic graphite (EPPG)), or nanomaterials including graphene and its derivatives [13,14]. Carbon-based electrodes enable the application of a broader range of positive potentials and are cheaper than gold. It should also be noted that carbon electrodes were used in vivo studies, e.g., as carbon fibers for neurotransmitter analysis in the brain [15]. Interchanging between gold and carbon surfaces poses a challenge in terms of the manner of bioreceptor attachment. Several approaches were tested, including the layer–by–layer (LBL) technique and electrode modification with polymers such as polyethyleneimine (PEI) or chitosan [16,17]. In such a case, nucleic acid immobilization is based on electrostatic attraction between the NA probe and the cationic layer placed on the electrode surface. However, this does not provide high stability of the receptor layer and might impede the efficiency of electron transfer. An interesting alternative might be the use of π–π interaction between carbon surface and nucleic acids, which can be conjugated with aromatic ring groups, including pyrene or anthracene, at the 5′ or 3′ end of nucleic acid [18,19]. The presence of aromatic rings might allow for the sustainability of receptor layers on carbon surfaces, and furthermore, such modification is limited to a one-step functionalization procedure.

For over twenty years, much interest has been given to electrochemical aptamer-based sensors (EABs) fabricated mainly on gold macroelectrodes and wires [20,21,22,23]. Those sensors usually contain methylene blue (MB)—modified aptamers, which undergo a conformational switch because of binding to the target molecule [24,25]. This led to the relocation of a label closer to the electrode surface (“signal on”) or its separation from the electrode (“signal off”), depending on the manner of conformational change. EABs were successfully employed for the detection of targets such as PDGF, cocaine, and vancomycin [26,27,28]. The latter is known as an antibacterial drug injected when skin or bloodstream diseases develop. So far, several examples of successful EAB elaboration for vancomycin in vitro and in vivo monitoring have been reported [29,30,31].

**Figure 1:**
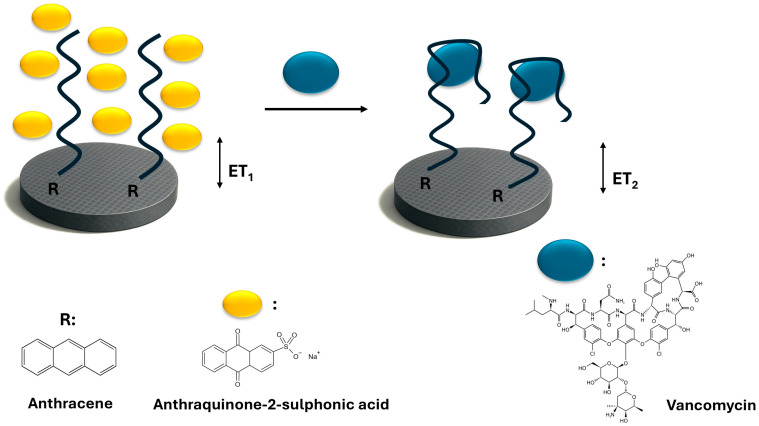
A representation of Vancomycin aptasensor performance.

Herein, we present studies on the application of carbon macroelectrodes (glassy carbon (GC) and edge-plane pyrolytic graphite (EPPG)) functionalized with a vancomycin aptamer via π–π interactions involving anthracene or pyrene anchors. The aptamers specific to vancomycin used in this research were identified before and applied for the functionalization of gold surfaces by the Plaxco and Arroyo-Currás groups [29,30,31,32,33]. The novel part is the application of one-step carbon surface modification, which was possible using an aptamer conjugated with pyrene/anthracene molecules. The studies were focused on the elaboration of the aptamer immobilization procedure, followed by selecting the redox indicator as the source of the aptasensor signal (Figure 1). This was continued by the determination of the dynamic response of the sensor as well as receptor layer selectivity and performance in a serum sample spiked with vancomycin. The research revealed that carbon electrodes could not only serve as an alternative transducer material for the effective elaboration of receptor layers through adsorption enhanced by π–π interactions but also enable vancomycin monitoring.

## 2. Materials and Methods

### 2.1. Apparatus

Electrochemical measurements, including cyclic voltammetry (CV), square-wave voltammetry (SWV), electrochemical impedance spectroscopy (EIS), and chronocoulometry, were conducted using CHI650 and CHI1040 potentiostats (CH Instruments, Bee Cave, TX, USA). The experiments were executed with application of a classical three electrode system including GC and EPPG (both from BASi, West Lafayette, IN, USA), serving as a working electrode, Ag/AgCl reference electrode (Mineral Polska, Łomianki-Sadowa, Poland), and gold wire (Merck, Darmstadt, Germany), which was used as a counter electrode. CV was executed within a potential range from −0.5 to 0.2 V in the presence of methylene blue, from −0.7 to 0.2 for ruthenium hexamine (RuHex), and from −0.7 to −0.2 V for a sodium anthraquinone-2-sulfonic acid (AQMS) indicator. SWV was executed within the same potential ranges with an amplitude of 0.025 V and a frequency of 15 Hz. Chronocoulometry measurements were conducted using potential between −0.4 and 0.1 V for a scan time of 0.5 s using RuHex. If necessary, EIS measurements were conducted at a dc potential of 0.2 V, an ac potential of 0.005 V, and a frequency range from 1 Hz to 100 kHz for ferri/ferrocyanide redox indicator.

### 2.2. Chemicals

Sodium dihydrogen phosphate, sodium hydrogen phosphate, magnesium chloride, potassium chloride, hexamineruthenium chloride, methylene blue, sodium anthraquinone-2-sulfonic acid, potassium hexacyanoferrate (III) (K_3_Fe(CN)_6_), potassium hexacyanoferrate (II) (K_4_Fe(CN)_6_), vancomycin hydrochloride, 2-phenoxyethanol, aspirin, kanamycin, streptomycin, doxycycline, and human serum from human male AB plasma were purchased from Sigma Aldrich (Saint Louis, MO, USA). Ethanol was purchased from POCH (Gliwice, Poland). Sodium chloride was purchased from Alfa Aesar (Waltham, MA, USA).

DNA aptamers (45 nt [32] and 66 nt [33]), containing anthracene or pyrene groups at the 5′ end, were synthesized and purchased from Metabion (Planegg, Germany) and listed in Table S1 (Supplementary Material). The aptamers were not conjugated to any redox-active labels. The most stable secondary structures of aptamers are shown in Table S2 (Supplementary Material). All sequences were stored as 100 µM stock solutions prepared in DNAase-free water and kept at −20° C.

### 2.3. Solutions

Following solutions were prepared: 20 mM PBS (20 mM NaH_2_PO_4_, 20 mM Na_2_HPO_4_, 150 mM NaCl, 2 mM MgCl_2_) pH = 7.4, 5 mM K_3_Fe(CN)_6_/K_4_Fe(CN)_6_ in 20 mM PBS, 5 mM K_3_Fe(CN)_6_/K_4_Fe(CN)_6_ in 100 mM KCl, 50 µM methylene blue in 20 mM PBS, 100 µM AQMS in 20 mM PBS, 50 µM RuHex in 20 mM PBS, 0.2 mM RuHex in 20 mM PBS, 2 mM 2-fenoxyethanol in 20 mM PBS, 1 mM vancomycin in 20 mM PBS, 1 mM aspirin in 20 mM PBS, 1 mM kanamycin in 20 mM PBS, 1 mM streptomycin in 20 mM PBS and 1 mM doxycycline in 20 mM PBS, 10% serum in PBS.

### 2.4. Electrode Cleaning and Functionalization

GC and EPPG electrode surfaces were cleaned by mechanical polishing on microcloth pads (Buehler, Lake Bluff, IL, USA) using Al_2_O_3_ powders with grain sizes of 0.3 and 0.05 µm. After washing with distilled water, the electrodes were sonicated at room temperature in H_2_O: ethanol solution (1:1, *v*/*v*) for 5 min. This was followed by measurements using CV obtained in 5 mM ferri/ferrocyanide in 0.1 M KCl with a scan rate of 0.1 V·s^−1^ for a potential range from −0.2 to 0.6 V, 2 cycles. If the electrodes’ purity was sufficient, regarded as the E difference between oxidation and reduction peaks did not exceed 120 mV, the electrodes were functionalized for 30 min. incubation with 2 µM aptamer (in 20 mM PBS) by dropcasting, and if necessary, this was followed by incubation with 2 mM blocking agent (2-phenoxyethanol).

Chronocoulometry measurements estimated the aptamer layer surface density on GC electrodes according to the protocol described in [34] using the equation:

$$\Gamma_{apt}=\;\frac{\left(Q_{Ru}-Q_{PBS}\right)}{nFA}\;\frac{z}{m}$$

where Γ_apt_ refers to the surface coverage of aptamer (mol·cm^−2^), *n* is the number of electrons involved in the redox process, F—Faraday constant, A—electrochemical surface of GC electrode, m is the number of bases in the probe sequence (45) and z (3) is the charge of ruthenium hexamine cation. Q_Ru_ (0.2 mM Ru(NH_3_)_6_^3+^) and Q_PBS_ (pure PBS) were obtained by referring to the relation of charge versus square root of time and estimating the intercept values by linear fitting of the curves obtained in 20 mM PBS or 0.2 mM Ru(NH_3_)_6_^3+^ in 20 mM PBS. Surface coverages of aptamer layers formed on GCE are presented in Table S3 (Supplementary Material).

### 2.5. Thioflavin T Strand Displacement Assay

Fluorescence studies were conducted using BioTek Synergy MX Microplate Reader (Agilent Technologies, Santa Clara, CA, USA) equipped with Gen5 software ver. 2.00.18. Firstly, 400 nM aptamer and 4 µM Thioflavin T solutions prepared in PBS were incubated for 40 min, which was followed by 40 min incubation with vancomycin, doxycycline, and kanamycin solutions. The fluorescence signal was measured (excitation at 425 nm, and emission at 495 nm).

### 2.6. AFM Imaging

AFM measurements were carried out using a Multimode 8 microscope controlled by a Nanoscope V controller (Bruker, Billerica, MA, USA). Aptamer samples were deposited from a PBS solution onto freshly cleaved HOPG substrates and incubated for 30 min to allow adsorption. The substrates were subsequently rinsed with Milli-Q water and dried under ambient conditions. Fresh HOPG surfaces were obtained by mechanical exfoliation with Scotch™ tape immediately before use. The substrates were mounted on metallic sample discs using adhesive tape. Topographical imaging was performed in PeakForce Quantitative Nanomechanical Mapping (PF-QNM) mode at room temperature using ScanAsyst-Air probes (Bruker, Billerica, MA, USA). All measurements were conducted under ambient laboratory conditions.

## 3. Results and Discussion

Vancomycin detection was often realized using electrochemical aptamer-based sensors (EABs). Such an approach is beneficial in terms of the limitation of chemicals applied during the measurement procedure, along with the number of steps required for the execution of the analysis using EABs. In the presented studies, we decided not only to test alternative transducer surfaces, namely carbon electrodes, but also to determine whether vancomycin can be detected using soluble redox indicators, allowing for their interchange to achieve the highest aptasensor response.

### 3.1. Selection of Carbon Electrode, Anchor, and Aptamer Length

There are several examples of the application of vancomycin aptamer [28,29,30,31,33] for functionalization of gold electrodes for in vitro and in vivo antibiotic monitoring. To determine whether carbon can substitute for the Au surface in the fabrication of aptamer layers, we first focused on the type of carbon electrode to use as a transducer. Hence, the aptamers were deposited on GC and EPPG electrodes using the same protocol, and this was followed by incubation with vancomycin. As shown in Figure S1 (Supplementary Material), incubation with the Vanco_A_short aptamer (45 nt) containing an anthracene anchor led to a significantly higher increase in electron transfer resistance (R_ET_) on the GC surface compared to the EPPG surface (1700 versus 500 Ohm). This indicates more efficient aptamer attachment on the GC surface, which is why we focused on the GC electrode as the transducer material. Next, the type of aromatic group serving as an anchor for aptamer attachment was chosen based on EIS experiments. It is clearly seen in Figure S2 (Supplementary Material) that modification with an anthracene–based aptamer led to more significant surface blockage than in the case of using a pyrene anchor (R_ET_ of 1300 vs. 1700 Ohm). In addition to a less pronounced electron transfer increase for the EPPG electrode, further incubation with 1 mM vancomycin led to a minor change of R_ET_, as can be seen in Figure S3C. This indicates more efficient binding between the aptamer and vancomycin when the GC surface served as a transducer. This convinced us to focus on aptamer—modified GC electrodes.

Further studies were performed using solely anthracene aptamers and aimed at selecting the aptamer length that yielded the highest electrochemical response. As no notable charge-transfer difference was observed with a longer (66 nt) aptamer (Figure S3B, Supplementary Material), a shorter (45 nt) version was selected. For Vanco_A_short aptamer, a R_ET_ drop upon incubation with vancomycin was evidenced, which is caused by the smaller repulsion of anionic ferri/ferrocyanide indicator from the receptor layer. This is most likely caused by the compensation of the negative charge of the aptamer layer that binds to positively charged vancomycin. As a result, the charge transfer between ferri/ferrocyanide and the electrode surface is not impeded to such an extent as in the absence of vancomycin. The application of a shorter version of an aptamer is also justified by results obtained by Plaxco [22], which is evidenced through circular dichroism studies that a shorter version of the aptamer underwent a conformation change upon binding to vancomycin. Consequently, no alteration of the rate of electron transfer was observed for the longer (66 nt) aptamer. The studies conducted by Dauphin-Ducharme et al. [22] and Shaver et al. [32] showed the advantage of using a shorter aptamer (45 nt) aptamer over the longer aptamer (66 nt). The circular dichroism studies pointed to a more pronounced change in signal when the 45 nt aptamer was incubated with vancomycin. The reason for the negligible change in R_ET_ when the GC electrode was modified with a 66 nt aptamer, which was incubated with vancomycin, is most likely caused by limited access of the target analyte to the aptamer strand, as surface-tethered aptamers can be tangled, and this minimizes the effectiveness of binding between the aptamer and vancomycin. As the aptamer–vancomycin complex is not formed, the access of ferri/ferrocyanide redox indicator to the electrode surface does not change. This outcome, along with the fact of high affinity of the truncated version of aptamer (45 nt) towards vancomycin (K_d_ of 0.1 μM for free aptamer [22] as well as 45 μM in flowing blood [22] and 110 μM in serum when applied as sensing layers [32]) convinced us to employ Vanco_A_short aptamer for further experiments. The advantage of 45 nt aptamer was also shown when we conducted SWV measurements. As can be seen in square-wave voltammogram (Figure S4, Supplementary Material), the AQMS current drop was significant after electrode incubation with 1 mM vancomycin when 45 nt aptamer was applied in comparison to the 66 nt probe. This indicates better performance and sensitivity of the aptamer-based sensor with receptor layer composed of shorter aptamer. We also aimed to compare K_d_ values for both aptamers using ThT strand displacement assay [35]. It enabled us to verify the binding affinities of both aptamers against vancomycin concentrations between 0.005 and 1 mM and presented that as a relation of relative Thioflavin T fluorescence versus target analyte concentration (see Figure S5, Supplementary Material). Based on the results we calculated dissociation constant values (K_d_), which were 0.003 and 0.055 mM for shorter (45 nt) and longer (66 nt long) aptamers, respectively. A lower Kd value indicated a higher affinity of the 45 nt aptamer for vancomycin compared to the 66 nt aptamer.

AFM analysis was employed to assess the formation of the sensing interface on the HOPG substrate (Figure S6). Following immobilization of the anthracene-functionalized aptamers, significant changes in surface topography were observed relative to bare HOPG. The resulting biomolecular film consisted of a homogeneous network-like layer with an average thickness of approximately 2.5 nm. No noticeable defects or uncovered regions were detected, indicating efficient aptamer immobilization and uniform surface coverage.

### 3.2. Methylene Blue (MB) vs. Sodium Anthraquinone-2-Sulfonic Acid (AQMS) Redox Reporter

The studies focused on selecting a redox indicator that generates an analytical signal through an oxidation/reduction process. Attempts were made using the following redox-active compounds [36]—positively charged: methylene blue (50 µM), hexamineruthenium chloride (50 µM), and negatively charged sodium anthraquinone-2-sulfonic acid (100 µM). The aptamer-modified GC electrodes were first incubated for 5 min in a redox indicator solution, followed by CV and SWV measurements. Next, a 10 min incubation with 1 mM vancomycin was performed by dripping it onto the electrode surface. The CV and SWV measurements were then repeated. This enabled the determination of the effect of the analyte on the biosensor response in the presence of the selected redox indicator. As can be observed in Figure S7 (Supplementary Material), a significant decrease in oxidation peak was observed when methylene blue or AQMS were present in the solution. Surprisingly, no signal change was observed when we tested RuHex [37]. This could be explained by the dominant role of electrostatic attraction between the positively charged ruthenium hexamine and the negatively charged aptamer. Such an interaction was not altered by vancomycin binding to the aptamer, indicating that the sugar-phosphate backbone remains exposed to the outer part of the receptor layer. Some of the RuHex molecules that are directly bound to the receptor layer could be repulsed upon aptamer binding to the target analyte. In such cases, the redox process can be a mixture of diffusion—controlled and surface—confined process. RuHex molecules that are in solution and are not bound directly to the aptamer layer can play a vital role, and the intensity of the signal is not affected by the aptamer–vancomycin interaction.

Next, we focused on the application of methylene blue and AQMS electroactive species. Their common feature is the presence of a sequence of aromatic rings, which enable hydrophobic or Π–Π interactions. Apart from that, electrostatic interactions play a substantial role in the binding between the MB/AQMS redox reporters and the aptamer–sensing layer. As those molecules differ in charge, a distinct response might be expected. To analyze the relation between aptasensor response versus vancomycin concentration, the signal change was defined as follows:

(1) $$Aptasensor\;response=\frac{I_{0}-I}{I_{0}}$$

where I_0_ and I refer to the current signal before and after electrode incubation with vancomycin, respectively.

**Figure 2:**
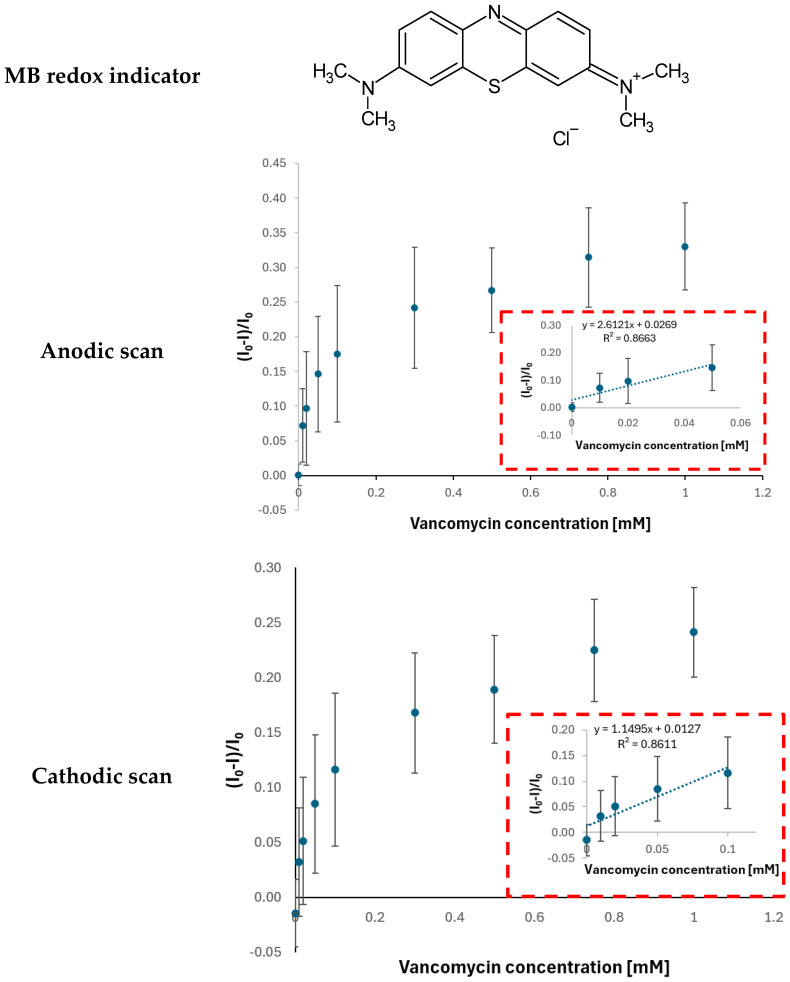
Relation of aptasensor response versus vancomycin concentration recorded using anodic and cathodic SWV scans in the presence of MB redox indicator.

As can be seen in Figure 2, a signal increase was observed both for anodic and cathodic scan direction when MB was present in the solution. However, across all tested concentrations, a significant standard deviation was observed, indicating limited repeatability of the sensor. The reason could be methylene blue adsorption on the GC surface, which was unoccupied by the anthracene–modified aptamer.

**Figure 3:**
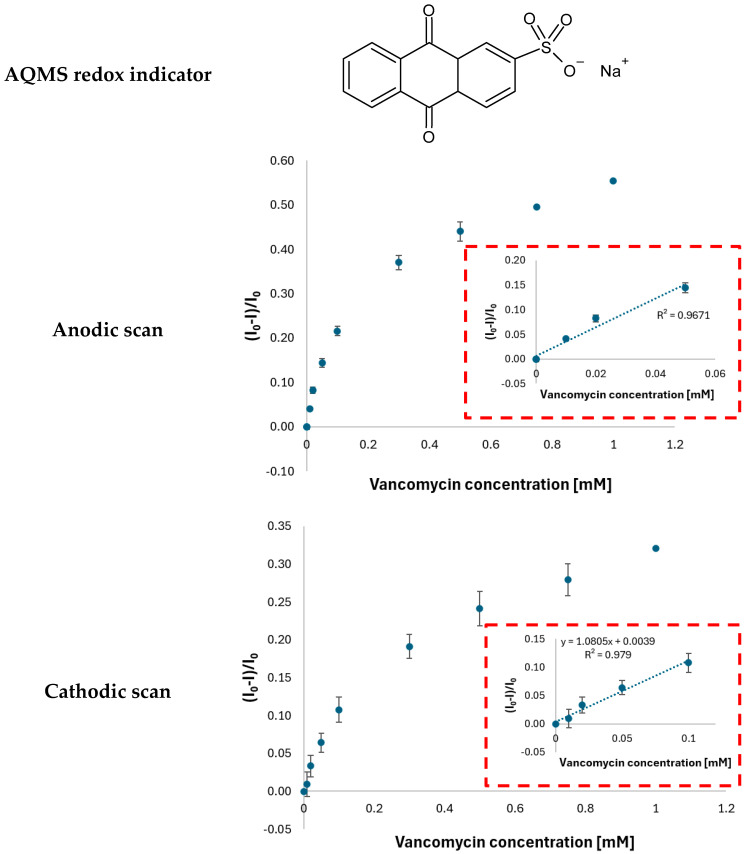
Relation of aptasensor response versus vancomycin concentration recorded using anodic and cathodic SWV scans in the presence of AQMS redox indicator.

Similar studies were performed for the AQMS redox indicator presented in Figure 3. Interestingly, both anodic and cathodic responses were distinguished with smaller standard deviations than those observed in experiments with MB. Furthermore, the signal change was more substantial for anodic responses. The aptasensor response increased from 0.01 to 1 mM vancomycin, which covers the range when applied in clinical approaches, including overdose monitoring, drug accumulation in patients with impaired renal function, pharmaceutical formulation and quality-control analysis, and characterization of local drug-delivery systems such as hydrogels, implant coatings, wound dressings, or bone cements, where vancomycin concentrations can reach high micromolar or millimolar levels. The lower limit of detection calculated using the following equation was 2.74 µM:

(2) $$LOD=\frac{3*SD}{a}$$

where SD refers to the standard deviation for the blank sample, and a is the slope for the linear part of the relation of aptasensor response versus vancomycin concentration.

### 3.3. Application of AQMS as a Redox Reporter

**Figure 4:**
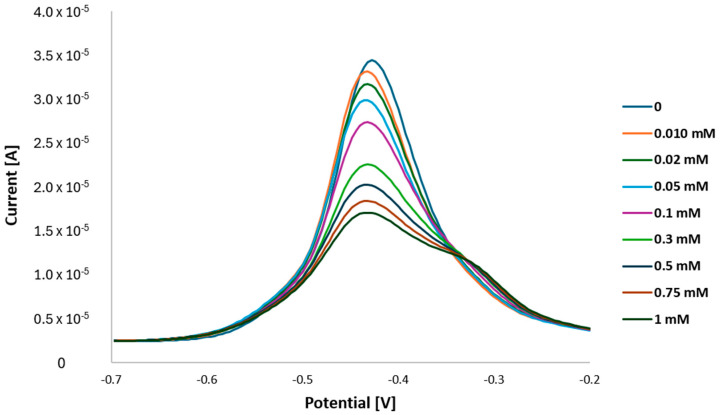
Square–wave voltammograms recorded for aptamer-modified GCE upon addition of vancomycin and in the presence of AQMS redox indicator.

When AQMS was used as a redox reporter, an anodic current decrease was observed upon addition of vancomycin (see Figure 4). For vancomycin concentrations exceeding 100 µM, a double oxidation peak was observed. Since vancomycin exhibited no oxidation signal at the bare GCE under the applied conditions, the additional oxidation peak cannot be attributed to its direct electrochemical oxidation. Instead, the second peak is likely associated with AQMS molecules present in different interfacial environments. The first oxidation peak corresponds to AQMS interacting with the aptamer layer via non-covalent interactions, while the other peak can be associated with AQMS interacting directly with exposed graphitic domains of the GCE [38]. Moreover, the aptamer sensing layer may not form a perfectly compact film and can contain defects or holes, allowing AQMS to access the underlying GCE surface. These differences in the local environment and electron-transfer kinetics can lead to distinct oxidation potentials and, consequently, to the appearance of a double oxidation peak in SWV. Hence, AQMS may interact with the aptamer layer through non-covalent interactions and/or adsorb directly on exposed GCE regions due to imperfections in the sensing layer.

It should be emphasized that the aptamer sensing layer might not be oriented upright relative to the electrode surface, and probe bending might be enhanced through nucleobases adsorption onto carbon. Such an effect is likely due to the anthracene dimensions and the probe length. To minimize such an effect, we decided to introduce a blocking agent, namely 2-phenoxyethanol. This compound also contains an aromatic ring, which allows for the GC surface functionalization. The influence of 2-phenoxyethanol on the aptasensor response was compared for electrodes subjected to incubation ranging from 10 to 60 min, and for electrodes with a receptor layer containing solely aptamer strands. It can be observed in Figure S8A (Supplementary Material) that the use of mixed aptamer/2-phenoxyethanol does not affect the aptasensor response when the linear range (meaning the concentrations from 0.01 to 0.1 mM) of vancomycin concentrations was taken into consideration. For all tested cases, the determination coefficient (R^2^) was above 0.94, indicating a strong correlation between the current change and vancomycin level.

**Figure 5:**
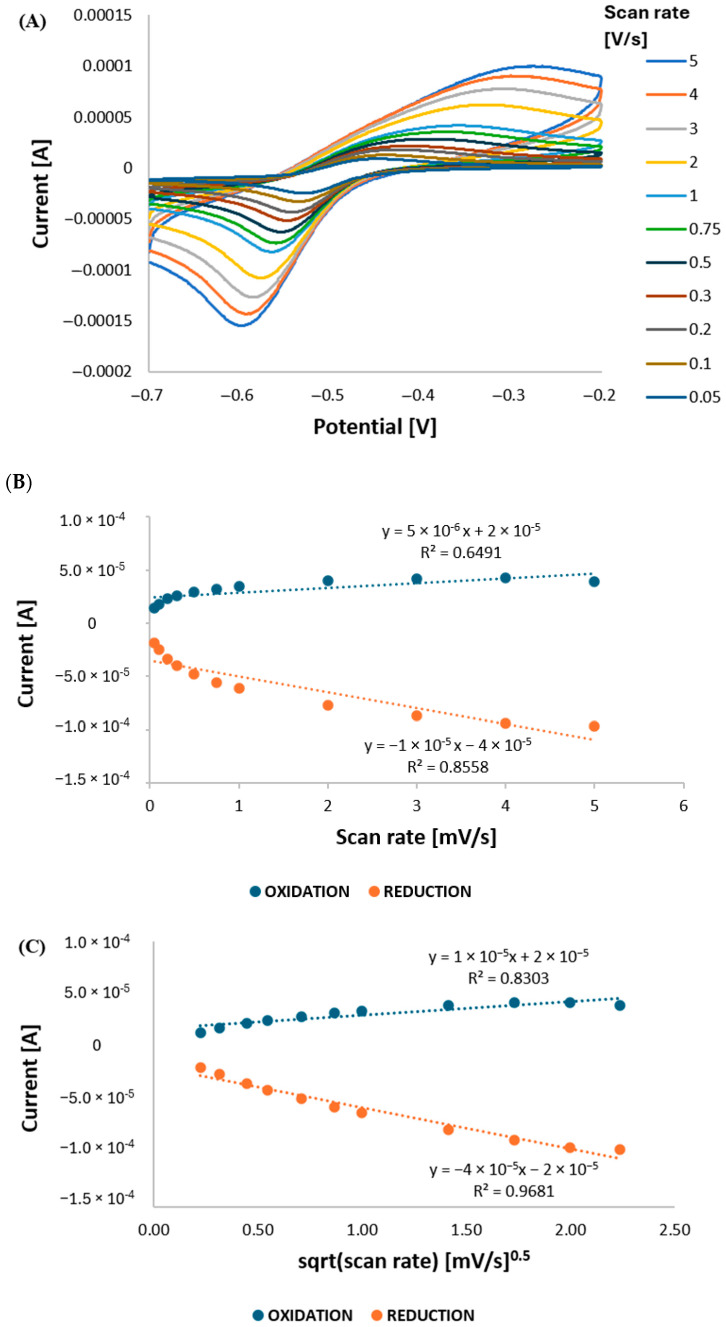
(**A**) Cyclic voltammograms recorded for the aptamer-modified GC electrode for various scan rates. (**B**) Relation of current versus scan rate and (**C**) square-root of scan rate.

Further electrochemical characterization of the redox process occurring on the GC surface evidenced a diverse appearance of oxidation and reduction responses for the AQMS redox indicator. As shown in Figure 5A, the anodic peak is much broader than the reduction peak. Moreover, the intensity of reduction currents is higher than for oxidation responses, which can be attributed to the occurrence of an irreversible redox process, and the possibility of AQMS surface adsorption as was observed in Figure 4. When currents were plotted against scan rate and the square root of scan rate (Figure 5B and C, respectively) it was noticed that the determination coefficient was higher for the latter. Such a result evidences the prevailing role of diffusion in the redox process, which is also likely because of the redox probe’s high concentration (100 µM) [39]. Similar behavior, namely a diffusion–controlled process, was observed for the GC prior and after modification with the anthracene-based aptamer (Figure S9, Supplementary Material).

### 3.4. Selectivity, Nonspecific Surface Adsorption and Serum Analysis

One of the key features of any biosensor is sufficient selectivity, which enables its application for analyte detection in complex physiological fluids such as serum and blood. In this study, we first tested the aptasensor responses separately for aspirin, kanamycin, streptomycin, and doxycycline, and then compared them with vancomycin. As shown in Figure S10 (Supplementary Material), aspirin and streptomycin cause a negligible response within 0.01–0.5 mM concentrations. On the contrary, a significant change in the aptasensor signal was recorded for doxycycline. This could be explained by the fact that doxycycline is smaller than vancomycin and might adsorb on the electrode surface. The direct binding of the doxycycline to the vancomycin aptamer is rather unlikely to happen [40], just like electrostatic attraction, since the doxycycline net charge at pH 7.4 is neutral. Interestingly, an adverse tendency of current change was observed for kanamycin, indicating an AQMS current increase upon analyte addition. This could be explained by the nonspecific adsorption of kanamycin onto the carbon surface, followed by electrostatic attraction to the negatively charged indicator. The latter effect is possible, as kanamycin is positively charged at pH 7.4.

We applied a ThT strand displacement assay to evaluate the binding affinity between aptamer and interfering molecules, which were doxycycline and kanamycin. Based on the results we calculated dissociation constant values (Kd), and in the case of doxycycline the relation of relative fluorescence versus doxycycline concentration did not resemble the adsorption curve, hence, it was not possible to calculate Kd value. This shows that the interaction between doxycycline and aptamer is not as typical as the one observed for vancomycin. The change in aptasensor response in the presence of doxycycline is therefore not a true target binding. Finally, for kanamycin the dissociation constant was 1.3 mM, which again shows significantly lower affinity of this drug towards vancomycin.

**Figure 6:**
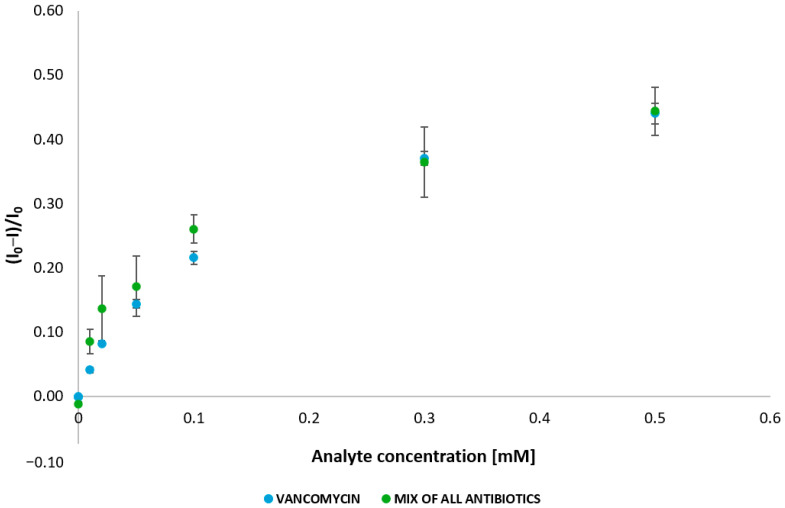
Aptamer-modified GCE response upon addition of vancomycin and a mixture of vancomycin and interfering antibiotics.

Next, the aptasensor was tested in a mixture of all antibiotics. It is clearly seen in Figure 6 that the biosensor response is similar when PBS containing solely vancomycin or the mixture of antibiotics was applied. Such a result suggests a minor effect of kanamycin or doxycycline, as well as a high affinity of vancomycin towards the aptamer. The negligible influence of interfering antibiotics is most likely due to vancomycin–aptamer complex formation, leading to rearrangement of the aptamer sensing layer. Consequently, the interferents might not adsorb onto the electrode surface.

Finally, the sensor response was checked in a 10% serum sample, both unspiked and spiked with 1 mM vancomycin. As shown in Figure S11 (Supplementary Material), the addition of vancomycin increased the signal during square-wave anodic scans. The aptasensor response was significant for unspiked serum, and this could also lead to impeding the current change when vancomycin was introduced. Consequently, the net aptasensor change is four times smaller than when PBS containing vancomycin was analyzed. Though the sensitivity of the aptasensor worsened, vancomycin could still be detected in a complex sample, and this proves the utility of the developed platform.

To conclude the studies, we verified the biosensor’s performance after 3 days of storage in PBS at 4 °C (Figure S12, Supplementary Material). The results obtained demonstrate the high potential for application of the prepared biosensor following the storage phase. The biosensor responses are lower, and the standard deviations are higher; however, the receptor layer did not lose its ability to recognize the analyte following the storage phase.

## 4. Conclusions

In the presented studies, we aimed to challenge the application of gold transducers and applied glassy-carbon electrodes as surfaces for the development of aptamer layers. To achieve that goal, we introduced DNA aptamers containing anthracene and pyrene anchor groups at the 5′ end. Because anthracene provided a higher R_ET_ change, we used it for electrochemical studies and vancomycin detection. The advantage of the application of AQMS as a redox probe over MB and RuHex was evidenced, and a proposed sensor was enabled for vancomycin detection within 0.01 to 1 mM concentration. The introduction of 2-phenoxyethanol as a blocking agent had a minor influence on the enhancement of the aptasensor response. The aptasensor’s selectivity was confirmed by testing against a mixture of the target analyte and other antibiotics. Finally, the aptasensor allowed for vancomycin detection in a serum sample, though its sensitivity was affected. The main advantage of the aptamer-based sensor on the GC surface over the Au surface is the short functionalization time, as shown in Table 1.

Future studies should be aimed at verification of the possibility of aptasensor miniaturization using, e.g., planar carbon electrodes, as this could enable vancomycin non-invasive or minimally invasive in vivo monitoring.

## Figures and Tables

**Table 1:** Comparison of working parameters of aptamer-based sensors for vancomycin detection.

Aptamer-Based Sensing Surface	LOD	Linear Range of Response	Aptamer Layer Preparation Time	Reference
Planar gold electrode modified with thiolated methylene-blue-labeled aptamer	69 nM	0.07–10 µM	1.5–2 h	[33]
Nanoporous gold electrode functionalized with thiolated vancomycin aptamer	Not explicitly reported	1–100 µM	2–3 h	[30]
Gold microelectrode with truncated aptamer	Not explicitly reported	1–100 µM	2 h	[32]
GC electrode modified with anthracene-aptamer	2.74 µM	0.01 to 0.1 mM	40 min.	This paper

## Data Availability

Dataset available on request from the authors.

## Supplementary Materials

The following supporting information can be downloaded at: https://www.mdpi.com/article/10.3390/bios16070353/s1, Figure S1: Nyquist plots presenting the effectiveness of immobilization of 2 μM Vanco_A_short aptamer on the surface of (A) GCE, (B) EPPG electrodes; Figure S2: Nyquist plots presenting the effectiveness of immobilization of (A) 2 μM Vanco_P_short aptamer (45 nt containing pyrene group) and (B) 2 μM Vanco_A_short aptamer (45 nt containing anthracene group) on the surface GCE; Figure S3: Nyquist plots presenting the effectiveness of immobilization of 2 μM anthracene—based aptamers containing (A) 45 nt (Vanco_A_short), (B) 66 nt (Vanco_A_long) on the surface GCE, and (C) 45 nt (Vanco_A_short) on the EPPG surface; Figure S4: Square-wave voltammograms recorded for GC electrode modified with 45 nt and 66 nt aptamers before and after incubation with 1 mM vancomycin. The responses were recorded using AQMS redox indicator; Figure S5: Curve presenting Relative ThT fluorescence versus vancomycin concentration derived from fluorescence experiments; Figure S6: AFM imaging for (a) bare HOPG and (b) HOPG after 30 min incubation with 2 µM anthracene—modified aptamer. (c) 3D AFM topography image of the HOPG after 30 min incubation with 2 µM anthracene—modified aptamer; Figure S7: A comparison of the SWV response of the aptasensor before and after incubation with 1 mM vancomycin in the presence of MB, AQMS, and RuHex redox indicators; Figure S8: (A) A comparison of aptasensor response versus vancomycin concentration for electrodes containing a mixed (aptamer/2-fenoxyethanol) layer and a vancomycin layer. (B) Comparison of GCE response containing receptor layer consisting of aptamer and mixture of aptamer and blocking agent (codeposition). The responses were derived from square-wave voltammograms (anodic scans) recorded using AQMS redox indicator; Figure S9: Relation of current versus scan rate (A,C) and square—root of scan rate (B,D) for GCE before (A,B) and after (C,D) modification with anthracene aptamer. All experiments were conducted in the presence of 100 µM AQMS; Figure S10: Aptamer—modified GCE response upon addition of vancomycin and interfering drugs. The responses were derived from square-wave voltammograms (anodic scans) recorded using AQMS redox indicator; Figure S11: Aptamer—modified GCE response 10% serum unspiked and spiked with 1 mM vancomycin. The responses were derived from square-wave voltammograms recorded using the AQMS redox indicator; Figure S12: A comparison of the biosensor’s response when measurements are taken immediately after the receptor layer has been formed and after 3 days of storage in PBS solution at 4 °C. The responses were derived from square-wave voltammograms recorded using the AQMS redox indicator; Table S1: A list of vancomycin aptamers; Table S2: Secondary structures of vancomycin aptamers along with ΔG values; Table S3: Aptamer surface coverages on GCE.

## Abbreviations

The following abbreviations are used in this manuscript:

AQMS	sodium anthraquinone-2-sulfonic acid
NA	Nucleic acid
DNA	Deoxyribonucleic acid
RNA	Ribonucleic Acid
PTO	Phosphorothioate
GC	Glassy carbon
EPPG	Edge-plane pyrolytic graphite
LBL	Layer-by-layer technique
PEI	Polyethyleneimine
EABs	Electrochemical aptamer-based sensors
MB	Methylene blue
PDGF	Platelet-Derived Growth Factor
RuHex	Ruthenium hexamine
PBS	Phosphate-Buffered Saline
