# Supplementary material for: Application of Aptamer–Carbon Surfaces for Electrochemical Label-Free Detection of Vancomycin
Source: Biosensors (Basel). 2026 Jun 24;16(7):353. doi: 10.3390/bios16070353 (PMC13407390; doi:10.3390/bios16070353)
Supplement: Supplementary file 1 [file biosensors-16-00353-s001.zip › biosensors-4346563-supplementary.pdf]

Application of aptamer – carbon surfaces for electrochemical label-free detection of vancomycin

Izabela Zaraś [1], Piotr Pieta [2], Marta Jarczewska [1,*]

1 Warsaw University of Technology, Faculty of Chemistry, Chair of Medical Biotechnology, Noakowskiego 3, 00-664 Warsaw, Poland; izabela.zaras.dokt@pw.edu.pl, marta.jarczewska@pw.edu.pl
2 Institute of Physical Chemistry Polish Academy of Sciences, Kasprzaka 44/52, 01-224 Warsaw, Poland

* Correspondence: marta.jarczewska@pw.edu.pl

Supplementary material

-

Table S1. A list of vancomycin aptamers.

Sequence name	5′ Anchor	Sequence (5′ – 3″)
Vanco_A_short	Anthracene	CGA GGG TAC CGC AAT AGT ACT TAT TGT TCG CCT ATT GTG GGT CGG [32]
Vanco_A_long	Anthracene	CTC TCG GGA CGA CCG AGG GTA CCG CAA TAG TAC TTA TTG TTC GCC TAT TGT GGG TCG GGT CGT CCC [33]
Vanco_P_short	Pyrene	CGA GGG TAC CGC AAT AGT ACT TAT TGT TCG CCT ATT GTG GGT CGG
Vanco_P_long	Pyrene	CTC TCG GGA CGA CCG AGG GTA CCG CAA TAG TAC TTA TTG TTC GCC TAT TGT GGG TCG GGT CGT CCC

Table S2. Secondary structures of vancomycin aptamers along with ΔG values.

Vancomycin sequence	Secondary structure	ΔG (kcal. mole^{-1})
**short**	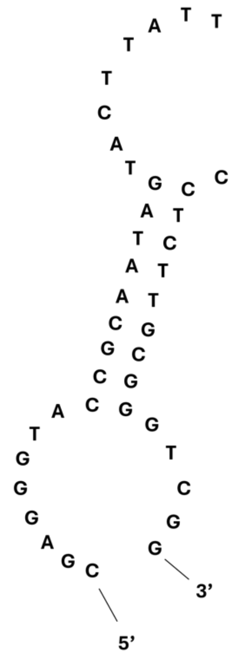	**-6.3**
**long**	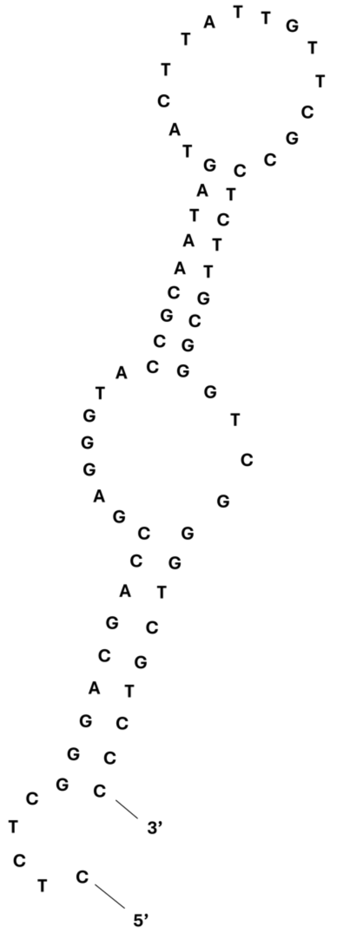	**-15.02**

Table S3. Aptamer surface coverages on GCE.

Sequence	Mean value [pmol·cm^{-2}]	SD [pmol·cm^{-2}]
Vanco_P_short	2.16	0.61
Vanco_A_short	2.30	0.50
Vanco_A_short + blocking agent	2.16	0.61

A)

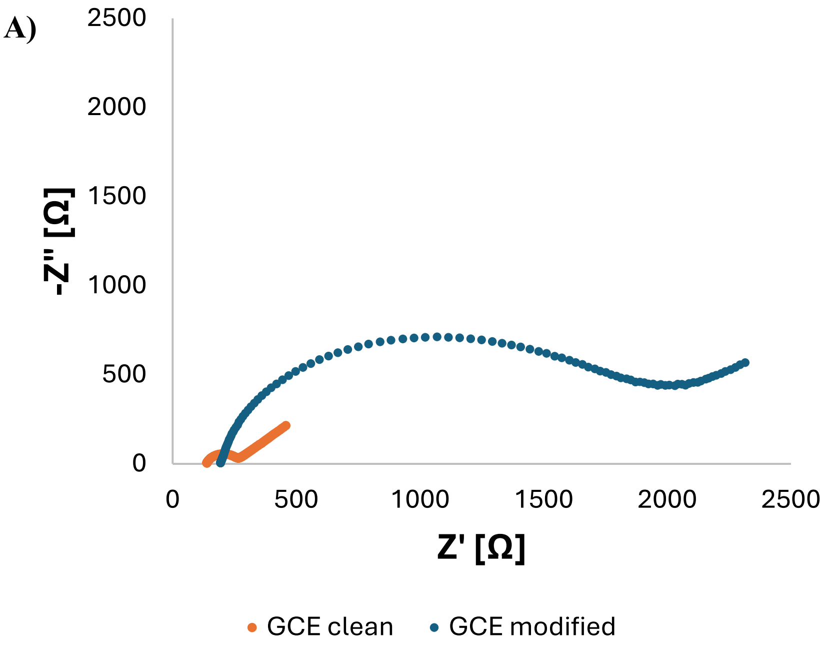

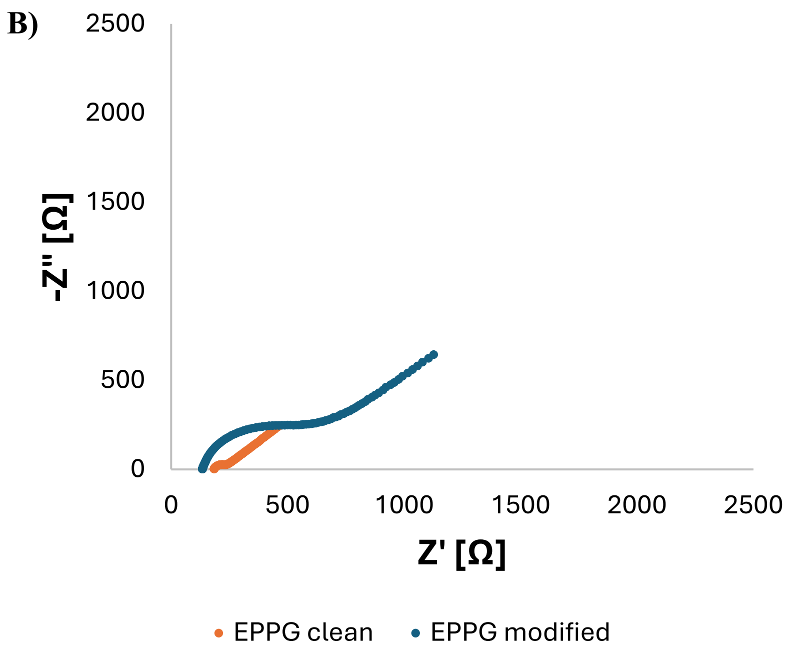

Figure S1. Nyquist plots presenting the effectiveness of immobilization of 2 µM Vanco_A_short aptamer on the surface of A) GCE, B) EPPG electrodes.

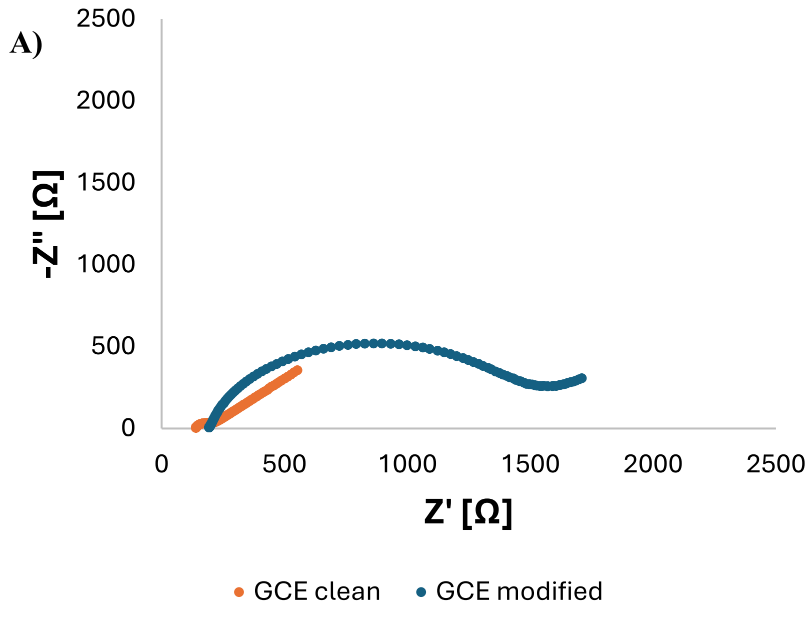

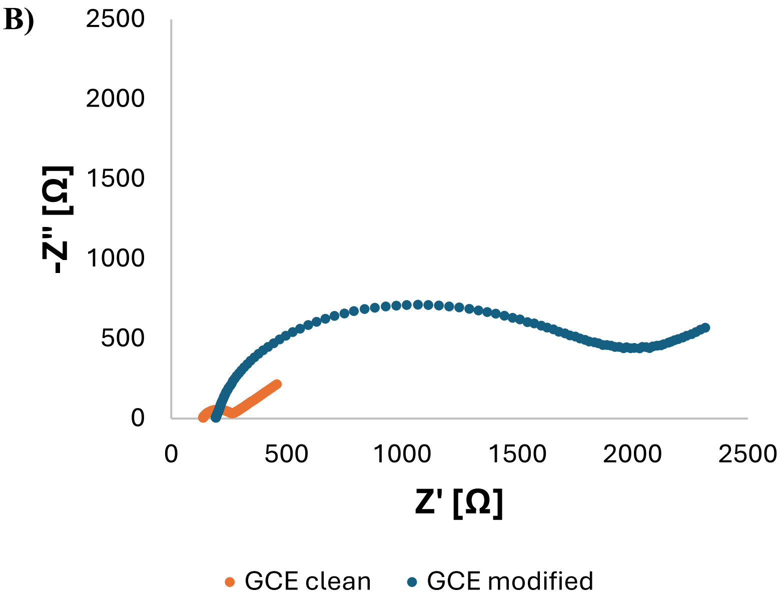

Figure S2. Nyquist plots presenting the effectiveness of immobilization of A) 2 μM Vanco_P_short aptamer (45 nt containing pyrene group) and B) 2 μM Vanco_A_short aptamer (45 nt containing anthracene group) on the surface GCE.

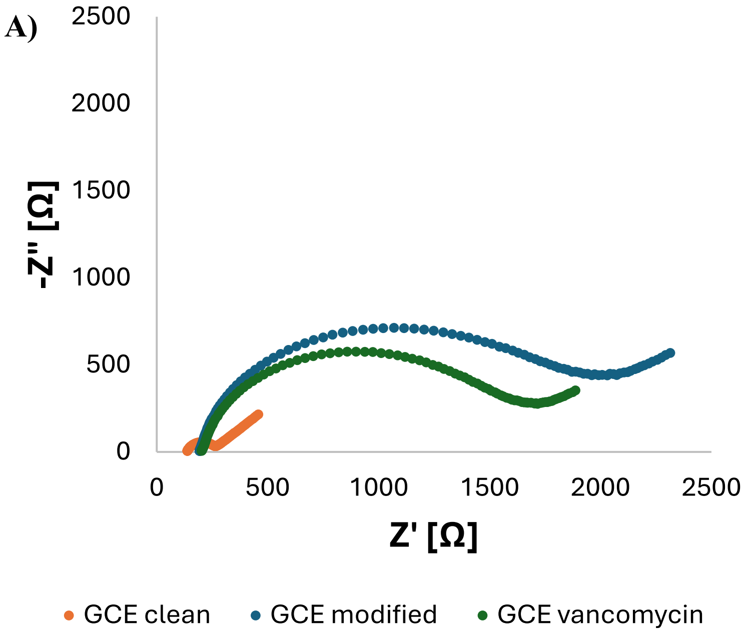

B)

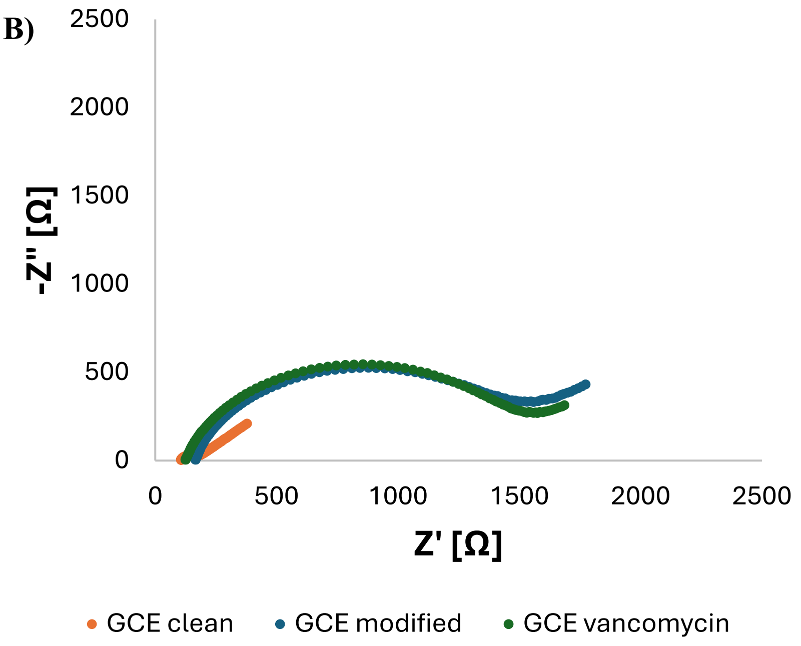

C)

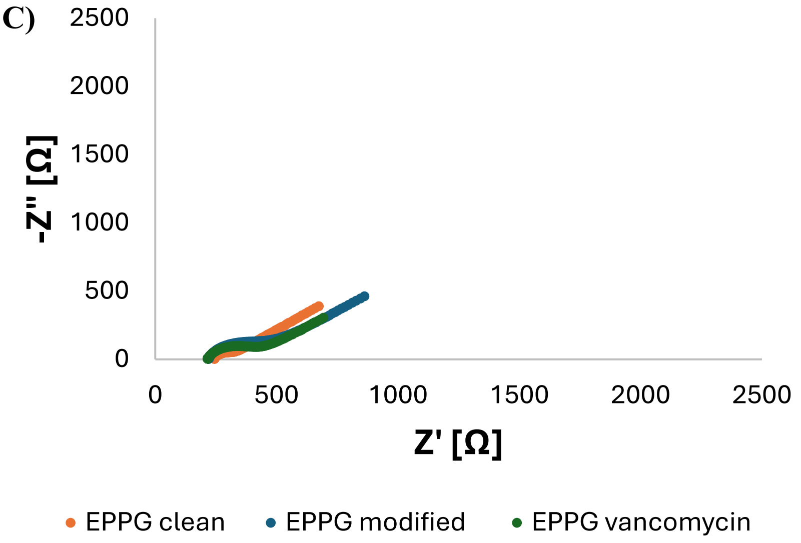

Figure S3. Nyquist plots presenting the effectiveness of immobilization of 2 μM anthracene – based aptamers containing A) 45 nt (Vanco_A_short), B) 66 nt (Vanco_A_long) on the surface GCE, and C) 45 nt (Vanco_A_short) on the EPPG surface.

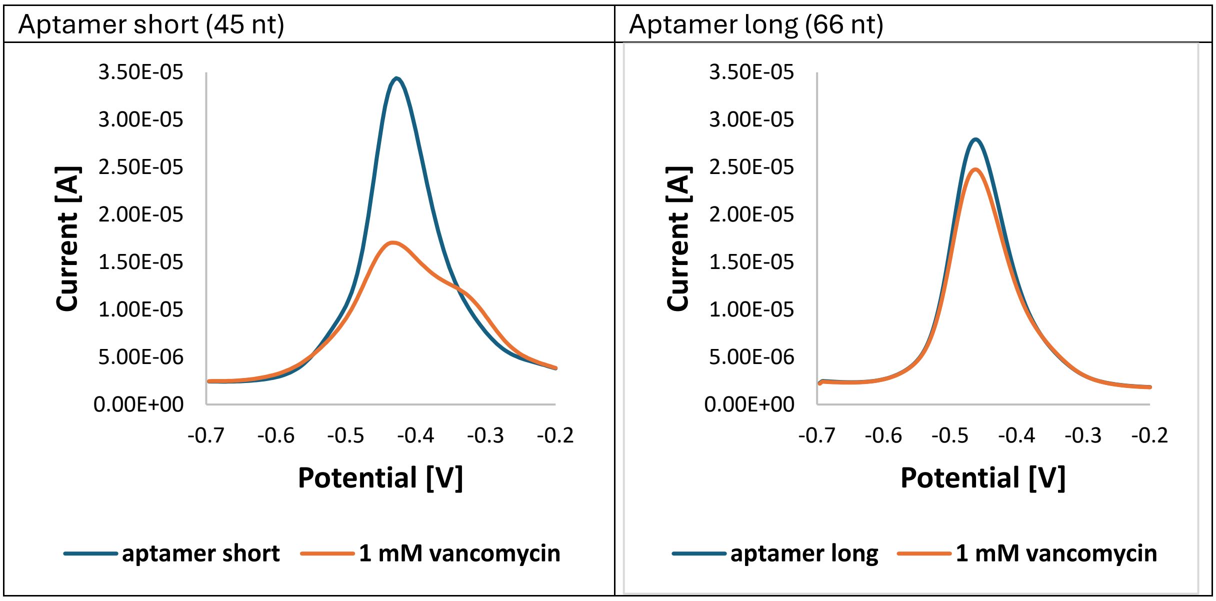

Figure S4. Square-wave voltammograms recorded for GC electrode modified with 45 nt and 66 nt aptamers before and after incubation with 1 mM vancomycin. The responses were recorded using AQMS redox indicator.

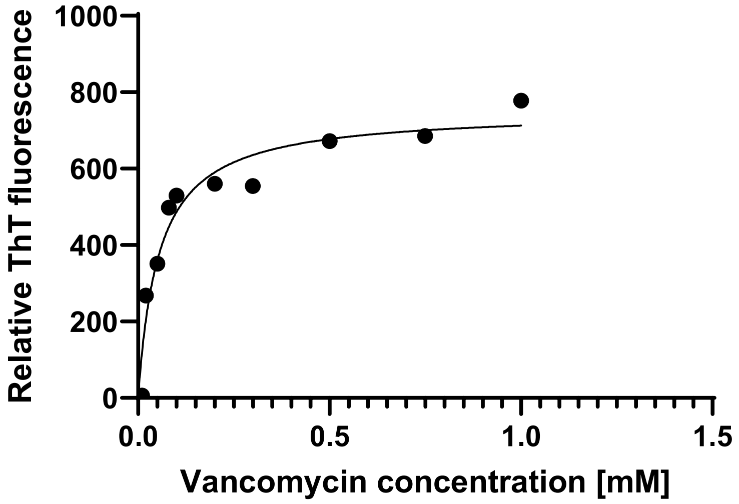

Figure S5. Curve presenting Relative ThT fluorescence versus vancomycin concentration derived from fluorescence experiments.

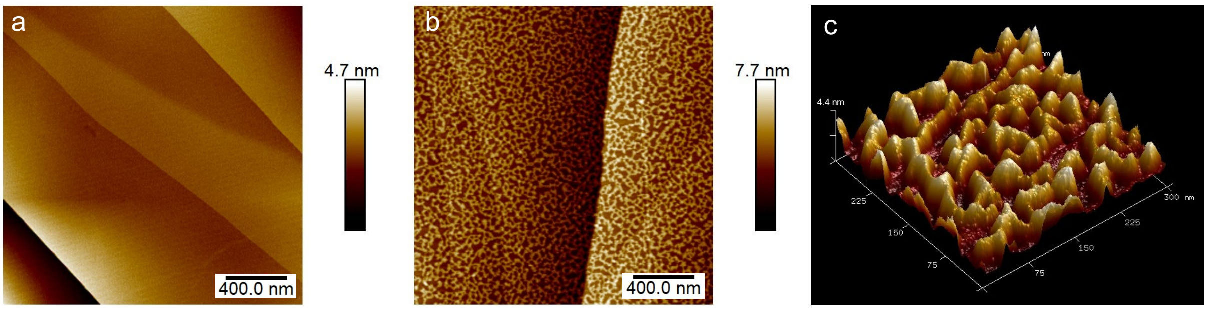

Figure S6. AFM imaging for (a) bare HOPG and (b) HOPG after 30 min incubation with 2 µM anthracene – modified aptamer. (c) 3D AFM topography image of the HOPG after 30 min incubation with 2 µM anthracene – modified aptamer

MB	AQMS	RuHex
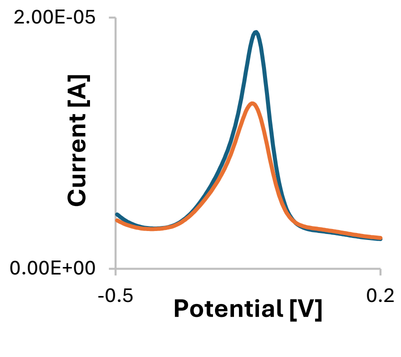	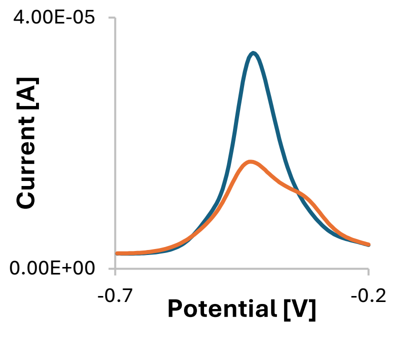	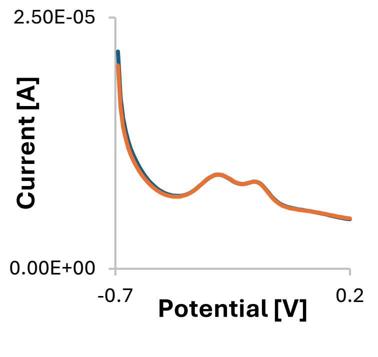

Figure S7. A comparison of the SWV response of the aptasensor before and after incubation with 1 mM vancomycin in the presence of MB, AQMS, and RuHex redox indicators.

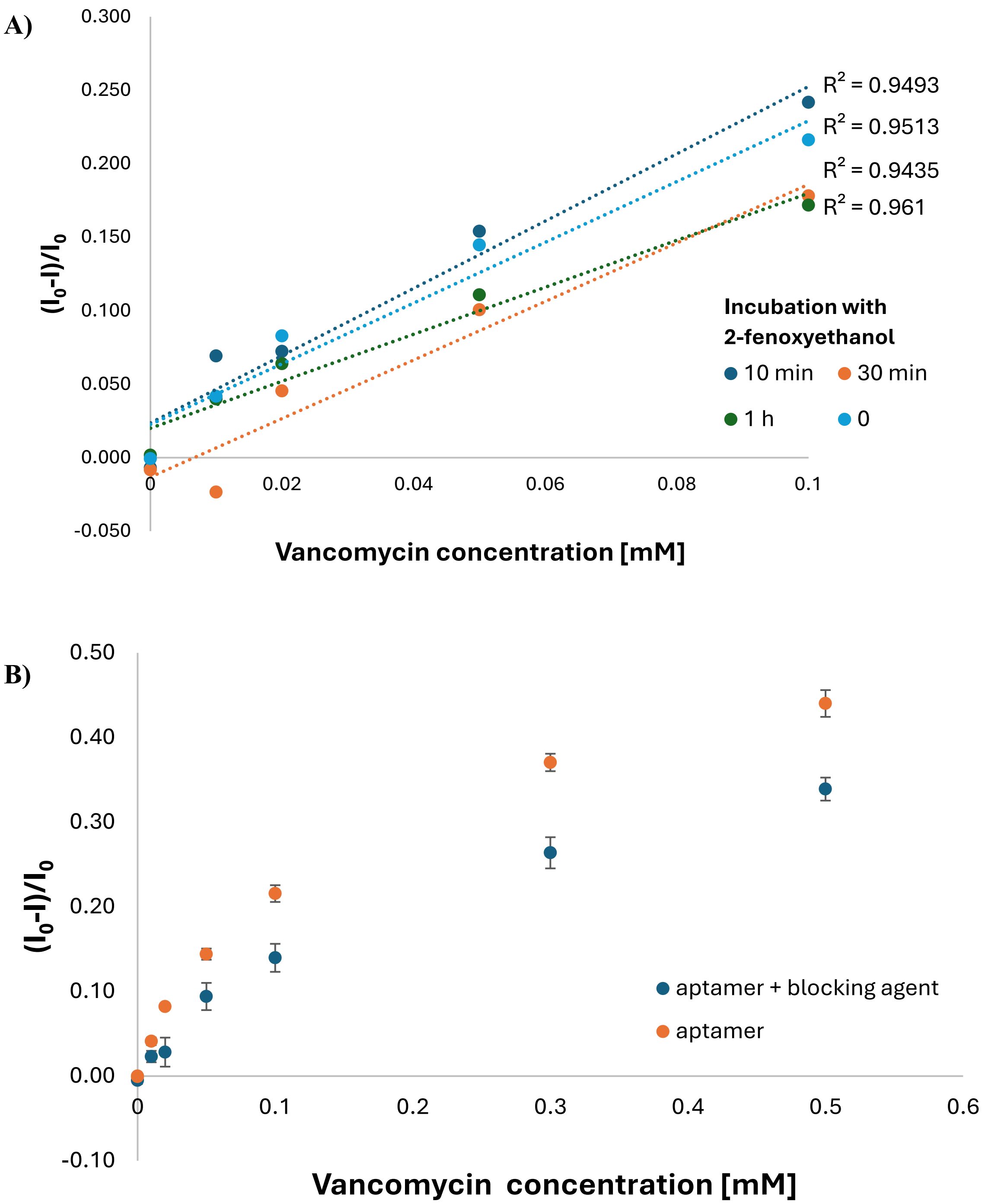

Figure S8. A) A comparison of aptasensor response versus vancomycin concentration for electrodes containing a mixed (aptamer/2-fenoxyethanol) layer and a vancomycin layer. B) Comparison of GCE response containing receptor layer consisting of aptamer and mixture of aptamer and blocking agent (codeposition). The responses were derived from square-wave voltammograms (anodic scans) recorded using AQMS redox indicator.

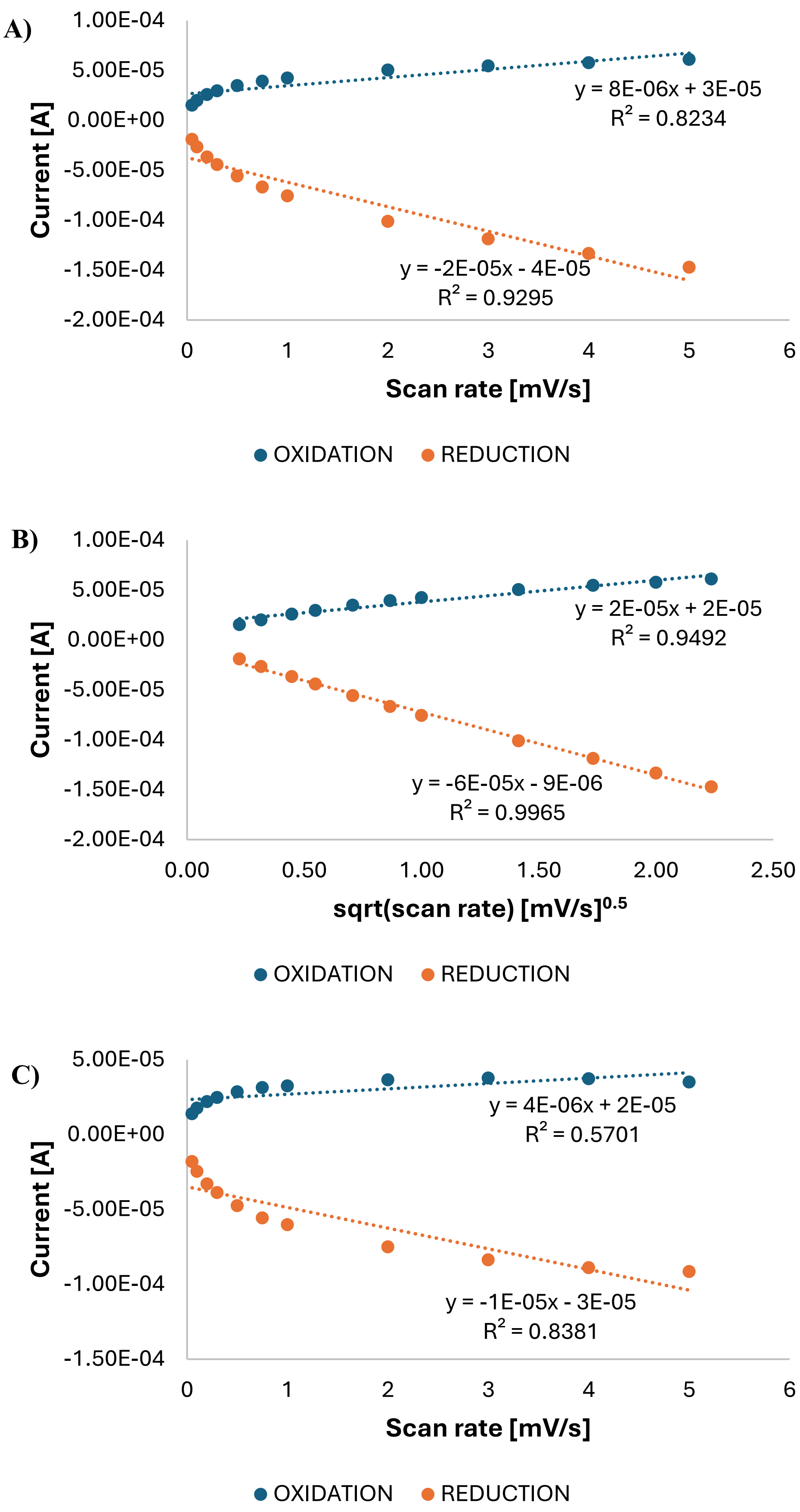

A)
1.00E-04
5.00E-05
0.00E+00
-5.00E-05
-1.00E-04
-1.50E-04
-2.00E-04
Current [A]
y = 8E-06x + 3E-05
R² = 0.8234
y = -2E-05x - 4E-05
R² = 0.9295
0
1
2
3
4
5
6
Scan rate [mV/s]
OXIDATION
REDUCTION
B)
1.00E-04
5.00E-05
0.00E+00
-5.00E-05
-1.00E-04
-1.50E-04
-2.00E-04
Current [A]
y = 2E-05x + 2E-05
R² = 0.9492
y = -6E-05x - 9E-06
R² = 0.9965
0.00
0.50
1.00
1.50
2.00
2.50
sqrt(scan rate) [mV/s]^0.5
OXIDATION
REDUCTION
C)
5.00E-05
0.00E+00
-5.00E-05
-1.00E-04
-1.50E-04
Current [A]
y = 4E-06x + 2E-05
R² = 0.5701
y = -1E-05x - 3E-05
R² = 0.8381
0
1
2
3
4
5
6
Scan rate [mV/s]
OXIDATION
REDUCTION

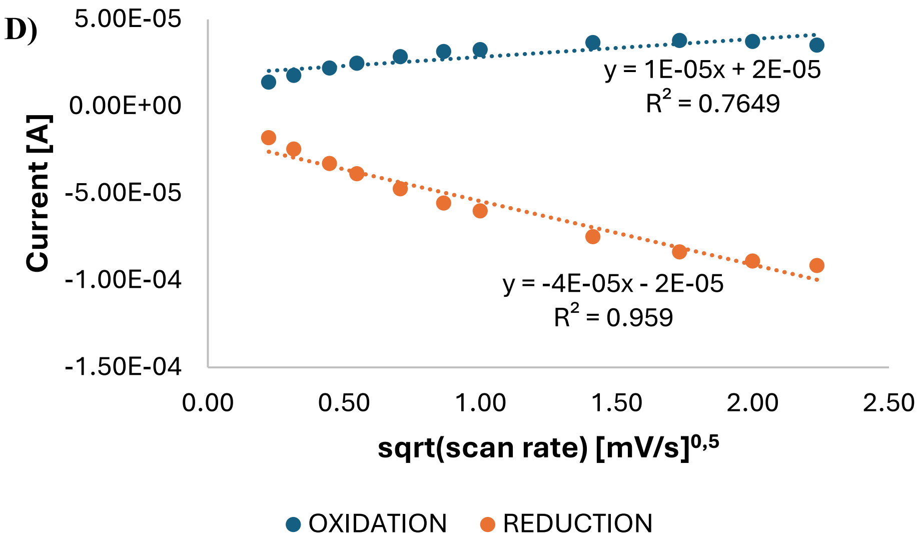

Figure S9. Relation of current versus scan rate (A, C) and square - root of scan rate (B, D) for GCE before (A, B) and after (C, D) modification with anthracene aptamer. All experiments were conducted in the presence of 100 µM AQMS.

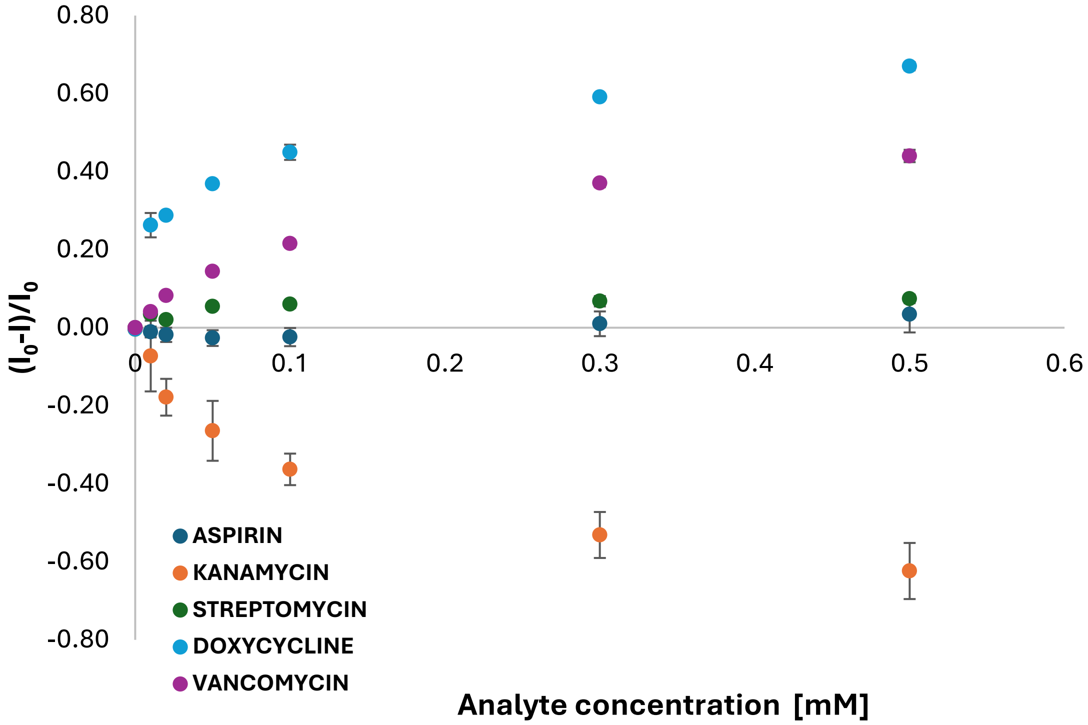

Figure S10. Aptamer – modified GCE response upon addition of vancomycin and interfering drugs. The responses were derived from square-wave voltammograms (anodic scans) recorded using AQMS redox indicator.

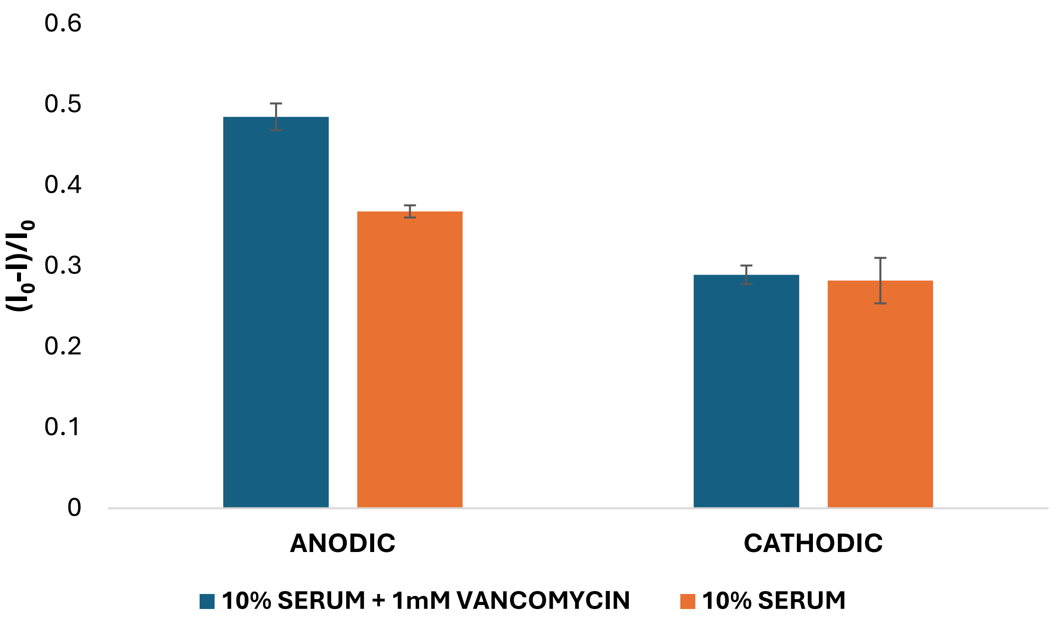

Figure S11. Aptamer – modified GCE response 10% serum unspiked and spiked with 1 mM vancomycin. The responses were derived from square-wave voltammograms recorded using the AQMS redox indicator.

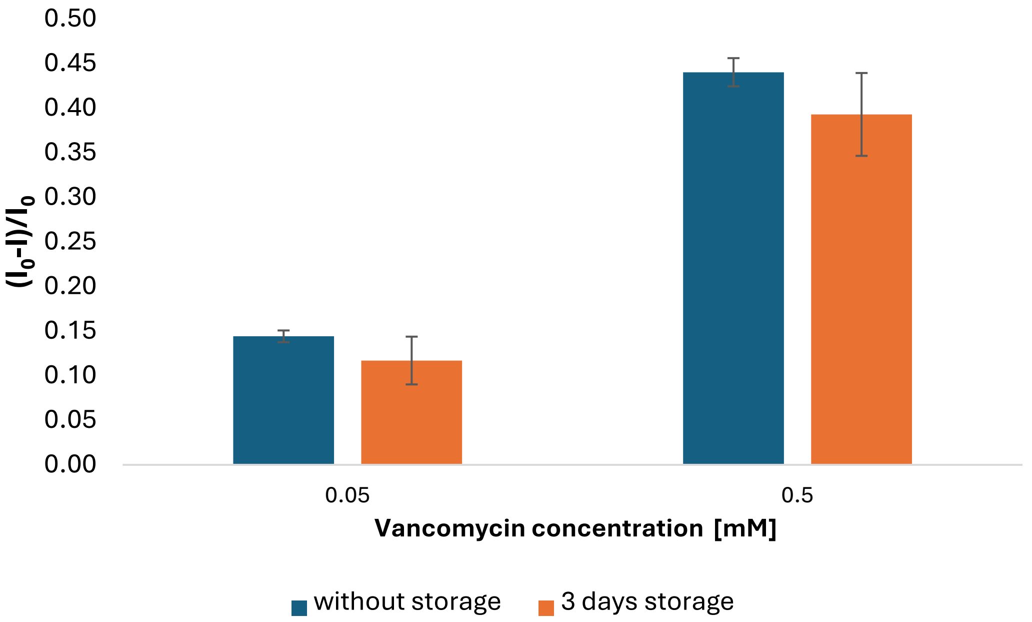

Figure S12. A comparison of the biosensor's response when measurements are taken immediately after the receptor layer has been formed and after 3 days of storage in PBS solution at 4 °C. The responses were derived from square-wave voltammograms recorded using the AQMS redox indicator.
